# Refractory Vasoplegic Syndrome in an Adult Patient with Infective Endocarditis: A Case Report and Literature Review 

**Published:** 2017-01

**Authors:** Seyed Mohsen Mirhosseini, Ali Sanjari Moghaddam, Paritash Tahmaseb Pour, Ali Dabbagh

**Affiliations:** Shahid Modarres Hospital, Shahid Beheshti University of Medical Sciences, Tehran, Iran.

**Keywords:** *Endocarditis*, *Syndrome*, *Vasopressins*

## Abstract

Postoperative vasoplegic syndrome (VS) is characterized by low systemic vascular resistance, normal or elevated cardiac output, and poor response to volume expansion. The incidence of VS after cardiac surgery requiring cardiopulmonary bypass is about 20%. Sometimes, VS becomes refractory and initial treatments do not work, rendering treatment a great challenge. In this study, we describe a young male patient with endocarditis undergoing tricuspid valve replacement. When being weaned off cardiopulmonary bypass, the patient experienced VS. The patient’s blood pressure did not increase after the administration of a high dose of epinephrine and norepinephrine. Therefore, he was commenced on a low dose of vasopressin and gradually his blood pressure reached the normal range. Although the standard management of VS is a high dose of vasopressors, this patient was refractory to a combination of epinephrine and norepinephrine; only a vasopressin infusion was able to treat the patient. Eventually, he was weaned from bypass and the operation was terminated satisfactorily. Thereafter, the patient passed the recovery period in the cardiac intensive care unit and was discharged. It seems that vasopressin is an excellent option in refractory vasoplegia with minimal response to other vasopressors.

## Introduction

Postoperative vasoplegic syndrome (VS) is characterized by low systemic vascular resistance, normal or elevated cardiac output, and poor response to volume expansion. The incidence of VS after cardiac surgery requiring cardiopulmonary bypass (CBP) is in the range of 5% to 25%.^1^^, ^^2^ High doses of vasopressors often require maintaining the adequate blood pressure postoperatively. Vasoplegia may become refractory to vasopressors and lead to high morbidity and mortality.^3^^, ^^4^ The most frequently used vasopressors are norepinephrine, vasopressin, and phenylephrine.^3^^, ^^5^

Here, we describe a male patient with tricuspid valve replacement due to endocarditis who experienced VS when being weaned off CPB. The patient’s hypotension was resistant to epinephrine and norepinephrine, but he displayed a dramatic response to vasopressin.

## Case Report

A 32-year-old man with a history of smoking (30 packs a year) and intravenous heroin injection for 5 years presented with productive cough, fever, malaise, and significant weight loss. He took no medication and had no history of prior disease. On examination, he was cachectic and a body temperature of 38.2 ˚C was recorded. Auscultation of the lungs revealed fine crackles and rhonchi in the base of the right lung. The remainder of the physical examination was unremarkable.

Laboratory tests showed neutrophil-dominant leukocytosis, high erythrocyte sedimentation rate (125 mm/h), and hemoglobin level of 8.5 g/dL. Chest X-ray demonstrated multiple cavities in both lungs. A computed tomography (CT) angiogram of the chest had no sign of septic pulmonary emboli. Further evaluation with a transthoracic echocardiogram revealed vegetation (30 × 25 mm in diameter) attached to the tricuspid valve, which favored acute infective endocarditis.

The patient was commenced on wide-spectrum antibiotic therapy and was administered treatment for endocarditis. Nevertheless, because of poor clinical improvement, he was considered for an emergent surgical operation. We requested a blood culture preoperatively and it was negative. We urged the patient to stop smoking and heroin injection and he wholeheartedly agreed. 

In the operating room, after anesthesia induction, CPB was conducted with 2.4 L/min/m^2^. Though the debridement of the infected areas and the excision of the vegetation was performed precisely, the tricuspid valve was unrepairable due to severe valvular damage. Therefore, the patient underwent tricuspid valve replacement with a biological tissue valve, which was performed satisfactorily. During the surgery, the mean arterial pressure (MAP) was in the normal range and the clamp time of the aorta was about 30 minutes. When being weaned off CBP, th patient’s blood pressure declined to 40 to 50 mmHg and the volume expansion was affected slightly. He was commenced on inotropes with a diagnosis of VS. He was initiated on an epinephrine drip at 0.02 µg/kg/min, which increased to 0.1 µg/kg/min. The MAP remained low, prompting us to add a norepinephrine drip at 0.02 µg/kg/min, which increased to 0.1 µg/kg/min. In addition, transesophageal echocardiography (TEE) revealed a normal myocardial and valvular function without any valvular complications. Despite receiving a high dose of epinephrine and norepinephrine, the patient’s MAP failed to rise significantly, while TEE demonstrated a good function for the left ventricle. Therefore, we added a vasopressin drip at 0.02 IU/min. Interestingly, the MAP gradually increased to 80-90 mmHg. We continued the vasopressin drip at 0.02 U/min, the norepinephrine drip at 0.02 µg/kg/min, and the epinephrine drip at 0.02 µg/kg/min.

The patient was transferred to the intensive care unit with empirical antibiotic therapy for septic shock. He was stable and after 24 hours, the vasopressors were discontinued. The result of the postoperative blood culture was positive for *staphylococcus aureus*. He was discharged 2 weeks later with no major surgical complications. The postoperative follow-up at 6 months revealed no complications.

## Discussion

To choose the best vasopressor drug for vasoplegia or VS after cardiac surgery, we performed a review of the literature; our search strategy included PubMed as our search engine (http://www.ncbi.nlm.nih.gov/pubmed/), searching for these key words: “methylene blue” and “vasoplegia” and “cardiac surgery”. No limit was used in the search. Finally our search, conducted from 2003 to 2014, resulted in 21 abstracts dealing with vasoplegia in cardiac surgery and also the use of 1of the 2 drugs: methylene blue or vasopressin

Though the number of studies on methylene blue was more than that of studies on vasopressin, the abstracts dealing with vasopressin were much more up to date and the trend of the studies demonstrated the superiority of vasopressin over methylene blue; this comparison between methylene blue and vasopressin is discussed here. Since vasopressin and methylene blue, 2 main vasopressor drugs proposed for the treatment of vasoplegia after cardiac surgery, are the major concern for this case, our discussion centers first and foremost around them. 

VS is considered among important topics in cardiac surgery, and vasopressin is a well-known vasopressor with well-known effects on VS; however, the disease is a relatively common condition after cardiac surgery requiring CBP. In our search, it was demonstrated that the occurrence of VS has a wide range, from 5% up to even 27% of all cardiac surgery patients; the difference in frequency between studies seems to be related to the differences in the populations under study in each of the articles, ranging from old-age populations undergoing coronary artery bypass graft up to those undergoing valve surgeries with or without infective endocarditis or even those with congenital heart disease.^2^^, ^^6^^-^^9^ Nearly all the studies dealing with the clinical outcome have demonstrated that the occurrence of the syndrome increases the chance for a poor clinical outcome or at least could deteriorate the clinical condition of the untreated patients. ^6^^, ^^10^^-^^14^ 

Vasoplegia is defined as intraoperative hypotension or postoperative hypotension (within 6 hours after weaning from CPB), decreased systemic vascular resistance (< 800 dynes/sec/cm^5^), low ﬁlling pressures, right atrial pressure < 5 mm Hg, left atrial pressure < 10 mm Hg, and normal or increased cardiac output (cardiac index > 2.5 L/min/m^2^). The main therapeutic goal in VS is to restore systemic vascular resistance to normal levels; both methylene blue and vasopressin have been shown to be able to achieve this goal.^6^^, ^^10^^, ^^15^^-^^17^ Also, nearly all these studies claim that vasoplegia after cardiac surgery is associated with a poor prognosis and has a high mortality rate. The administration of vasoactive drugs is always considered the cornerstone of standard management.^14^^, ^^16^^, ^^18^^-^^20^ 

Okamoto et al.^21^ demonstrated the safety and superiority of vasopressin in patients with catecholamine-unresponsive shock without any increase in the level of cardiac enzymes. Wittwer et al.^2^ demonstrated the efficacy of vasopressin in patients with vasoplegia suffering from right-sided congenital heart failure undergoing cardiac surgery. On the other hand, Cho et al.^16^ demonstrated that in patients with infective endocarditis undergoing heart valve surgery, prophylactic methylene blue had no effect on the total dose of vasopressor drugs. Lehot et al.^14^ presented a case report with vasoplegia after 3 hours of cardiac surgery with both methylene blue and vasopressin being administered while refractory asystole happened 5 hours after the operation; neither of the drugs could be beneficial in the final outcome. A study performed by Colson et al.^22^ is not only very interesting but also definitive insofar as the authors demonstrated that post-cardiac surgery vasodilation was the result of the depleted stores of vasopressin and defects in vasopressin stores after cardiac surgery. If we consider the details of their study, we could reach the conclusion that vasopressin is the treatment of choice for vasoplegia after cardiac surgery. Gilbert et al.^23^ enumerated the benefits of vasopressin for the treatment of vasoplegia after cardiac surgery and demonstrated the benefits of this drug compared with other agents. Lavigne^19^ in 2010 suggested both vasopressin and methylene blue as effective drugs; no superiority was given to either agent in that study. Elsewhere, Del Duca et al.^18^ presented 2 cases of vasoplegia after cardiac surgery, possibly due to aprotinin reaction, which were vasopressin refractory and responded to methylene blue.

Nevertheless, what mechanism could explain the pathophysiology of the disease? Although many mechanisms have been proposed for the disease, the etiology of VS is still speculative. ^1^^, ^^8^^, ^^13^  Long-term use of preoperative vasodilator medications, which leads to the accumulation of drugs in the tissues and the release of cytokines and inflammatory mediators; endothelial dysregulation, causing profound and refractory vasodilatation; use of vasodilators during CBP; preoperative cardiac dysfunction; and poor end-organ responsiveness after cardiac surgery likely explain the pathophysiology of VS. 4^, ^^9^^, ^^24^^-^^26^  Even, one of the studies has proposed that methylene blue could trigger apoptosis in endothelial cells; a phenomenon which is both dose dependent and time dependent.^27^

However, as was mentioned above, the very interesting study published by Colson et al.^22^ possibly describes the best plausible mechanism for vasoplegia after cardiac surgery, especially in patients who have received large doses of vasopressors or inotropes before surgery, which could be correlated with the superiority of vasopressin use over methylene blue or any other drug in such patients.

On the other hand, all of the above mechanisms might result in the activation of adenosine triphosphate-sensitive potassium channels (KATP channels) in the plasma membrane of vascular smooth muscles, activation of the inducible form of nitric oxide synthase, and deﬁciency of the hormone vasopressin, which can cause low vascular resistance.^10^ While vasopressin has its effects on the central nervous system and the vascular bed, it seems that the effect of methylene blue is mainly on the endothelium and smooth muscle cells of the vascular bed.^5^^, ^^7^^, ^^28^ The risk factors of vasoplegia include the preoperative use of angiotensin-converting enzyme inhibitors, calcium-channel blockers, and beta-blockers, as well as an increase in the duration of CPB, longer perfusion time, low preoperative ejection fraction, and inflammatory process such as septic shock.^1^^, ^^5^^, ^^29^ The systemic inflammatory response could be exaggerated in patients with infective endocarditis undergoing CPB,^4^^, ^^30^ so such patients have increased risk factors for the occurrence of vasoplegia possibly due to accumulated etiology of the inflammatory response.^3^^, ^^5^^, ^^10^^, ^^16^  We know that our patient denied taking medications and so, we could possibly assume that the most probable etiology of VS in our patient was the inflammatory response and septic shock due to endocarditis. Preoperative blood culture was negative and it is probably because of the first-stage medical management of the patient.

The ischemia of the distal portions of the upper and lower extremities as well as mesenteric ischemia is a complication of high-dose vasoconstrictor therapy.^31^ Therefore, preoperative determination of high-risk patients may increase the ability of the surgical team for the proper management of VS. On the other hand, the use of the least dose of vasopressors can reduce the complications. In the present study, the patient had a poor response to a high dose of epinephrine and norepinephrine but after adding low-dose vasopressin, his MAP increased to the normal range in a short time. The VS mechanisms to restore vascular tone include the activation of V_1_ vascular receptors in vascular smooth muscle cells, modification of adenosine triphosphate (ATP)-sensitive K+ channels (KATP), modulation of nitric oxide, and potentiation of adrenergic and other vasoconstrictor agents leading to vasoconstriction. ^1^^, ^^5^^, ^^32^  Recent literature suggests methylene blue and high-dose hydroxocobalamin for the refractory state of VS.^1^^, ^^10^^, ^^33^^, ^^34^ The results of our review discussed here suggest that vasopressin could be a worthy alternative to methylene blue or other vasoactive drugs in patients with refractory vasoplegia who do not respond to other vasopressors. Also, the trend of the above studies demonstrates that as long as we compare the 2 drugs, vasopressin seems much more superior to methylene blue-especially when considering the pathophysiologic basis for vasoplegia after cardiac surgery. Accordingly, vasopressin is suggested as the first-line vasopressor to treat vasoplegia after cardiac surgery.^3^

One of the noteworthy concerns about this patient was how we confirmed the diagnosis of vasoplegia after cardiac surgery. No pulmonary artery catheter (PAC) was inserted for the patient before skin incision since the patient was monitored with TEE. Recent studies have demonstrated the superior efficacy of TEE to the PAC in the assessment of loading conditions and patient outcome; we, therefore, utilized TEE rather than the PAC in our patient. ^35^^-^^37^ 

In light of the aforementioned, the question remains as to what is novel with the case presented herein? Firstly, we have focused on the etiology and diagnosis of the patient’s hypotension. Possibly, when considering the clinical course of the disease, we reach the conclusion that there is an important role for endocarditis in this patient. Given the clinical course of this patient, we come to the hypothesis that the cellular and molecular interactions induced to the patient by endocarditis have led to VS. Consequently, the present study may contribute to future studies on the assessment of the molecular and cellular mechanisms of the disease with a view to finding much more effective treatments bearing in mind that the current treatments simply constitute a symptomatic treatment of VS and as such fail to directly target the etiologic mechanism.^2^^, ^5^, ^^38^ 

## Conclusion

In refractory vasoplegia after cardiopulmonary bypass, when the clinical hypotension is refractory to different types of catecholamines, vasopressin can be considered an option for eliminating vasoplegia-associated hypotension.

## Notes:

***This paper should be cited as: ***Mirhosseini SM, Sanjari Moghaddam A, Tahmaseb Pour P, Dabbagh A. Refractory Vasoplegic Syndrome in an Adult Patient with Infective Endocarditis: A Case Report and Literature Review. J Teh Univ Heart Ctr 2017;12(1):27-31.
